# Safety, feasibility and efficacy of exercise as an airway clearance technique in cystic fibrosis: a randomised pilot feasibility trial

**DOI:** 10.1136/thorax-2025-223080

**Published:** 2025-10-01

**Authors:** Don S Urquhart, Emily Taylor, Steve Cunningham, Steff Lewis, Aileen Rae Neilson, Dia Soilemezi, Hannah Ensor, Ioannis Vogiatzis, Lorna J Allen, Zoe Louise Saynor, ExACT-CF Study Group, Debbie Miller

**Affiliations:** 1 Department of Child Life and Health, University of Edinburgh, Edinburgh, UK; 2 Department of Paediatric Respiratory and Sleep Medicine, Royal Hospital for Children and Young People, Edinburgh, UK; 3 Centre for Inflammation Research, University of Edinburgh, Edinburgh, UK; 4 Edinburgh Clinical Trials Unit, Usher Institute, University of Edinburgh, Edinburgh, UK; 5 Department of Psychology, Sport and Exercise Sciences, University of Portsmouth, Portsmouth, UK; 6 Department of Sport, Exercise and Rehabilitation, Northumbria University, Newcastle upon Tyne, UK; 7 Cystic Fibrosis Trust, London, UK; 8 School of Health Sciences, University of Southampton, Southampton, UK; 9 Wessex Cystic Fibrosis Unit, University Hospitals Southampton NHS Foundation Trust, Southampton, UK; 10 NIHR Southampton Biomedical Research Facility, University Hospital Southampton NHS Foundation Trust, Southampton, UK

**Keywords:** Cystic Fibrosis, Exercise, Paediatric Lung Disaese, Pulmonary Rehabilitation, Respiratory Function Tests

## Abstract

**Objectives:**

To test the feasibility and safety of exercise as an airway clearance technique (ExACT) for people with cystic fibrosis (pwCF) versus usual care (UC).

**Methods:**

Dual-site, two-arm randomised pilot trial. Fifty pwCF (≥10 years, forced expiratory volume in 1 s (FEV_1_) ≥40% predicted), stable on Elexacaftor/Tezacaftor/Ivacaftor, were recruited, of whom 48 were randomly assigned (1:1 with minimisation) to daily ExACT (stopping all other airway clearance techniques) or UC. Feasibility was measured by recruitment, retention and adherence against preset progression criteria. Key measures of safety and signals of efficacy included spirometry (FEV_1_), lung clearance index (LCI_2.5_), pulmonary exacerbations, physical activity, treatment burden and quality of life across 28 days. Qualitative interview data and preliminary health economic data were also collected.

**Findings:**

ExACT was safe over 28 days, measured by change in LCI_2.5_ (ExACT −0.1 (0.6) vs UC 0.2 (0.8), mean (SD)) and FEV_1_ (ExACT +2.1 (6.6) vs UC −0.8 (5.5), % predicted mean (SD)). Relative (ExACT/UC) differences of 0.97 (0.92, 1.02) for LCI_2.5_ and absolute differences (ExACT-UC) of 3.2 (−0.6, 6.9) % predicted for FEV_1_ suggest potential intervention efficacy. Few adverse events were reported; none serious. Recruitment and retention data suggest progression to a definitive trial, with 48/117 (41% of approached) randomised, 45/48 (92%) completing the study and a 60% overall adherence rate.

**Discussion:**

Testing of our primary hypothesis within a feasibility trial showed ExACT to be a safe, acceptable and feasible intervention for pwCF. These data support advancement to a definitive, longer-term, multisite trial evaluating the safety, efficacy and cost-effectiveness of ExACT, following minor refinement.

**Trial registration number:**

NCT05482048.

**WHAT IS ALREADY KNOWN ON THIS TOPIC**

- Chest physiotherapy airway clearance techniques (ACTs) are essential for people with cystic fibrosis (CF) but are time-consuming and burdensome. Intense aerobic exercise that incorporates forced expiratory techniques offers a promising alternative to ACT, but its safety and effectiveness need evaluation.

**WHAT THIS STUDY ADDS**

- People with CF were open to participating, and trial processes were feasible. Exercise as an ACT (ExACT) appeared safe and may be as effective as chest physiotherapy over 28 days, but a larger, longer trial is needed to definitively establish safety and efficacy.

**HOW THIS STUDY MIGHT AFFECT RESEARCH, PRACTICE OR POLICY**

- ExACT could ease treatment burden, offer greater flexibility and better align with patient preferences. A larger trial could confirm efficacy and, if established, subsequently demonstrate effectiveness and support its integration into CF care.

## Introduction

 Cystic fibrosis (CF) is an inherited disorder characterised by thick mucus accumulation in vital organs. Clearing secretions from the lungs through airway clearance techniques (ACTs), such as chest physiotherapy, combined with mucoactive agents, is essential to prevent recurrent infections and airway inflammation that may lead to airway obstruction and mucus plugging,^1^ airway damage, loss of respiratory function^2^ and eventual respiratory failure. ACTs employ various methods and devices to remove excess mucus secretions, aiming to alleviate airway obstruction and prevent respiratory tract infections, as well as to re-expand atelectatic areas, improve gas exchange and reduce inflammation.^3^

For over 60 years, performing regular chest physiotherapy has been central to CF care.^4^ Standard practice globally involves at least daily chest physiotherapy,^5^ increasing during exacerbations. To meet the diverse needs of people with CF (pwCF),^6^ chest physiotherapy as ‘usual care’ for airway clearance encompasses a variety of techniques, including positive expiratory pressure, oscillating devices, active cycle of breathing techniques and autogenic drainage. Despite the range of available options, chest physiotherapy is often performed with low quality.^7^ Many pwCF find it both time-consuming^8^ and burdensome,^9^ leading to poor adherence.^10 11^ The landscape of CF has changed with widespread availability of highly effective modulator therapy (HEMT), which reduces exacerbations^12^ and improves life expectancy,^13 14^ prompting exploration of alternative approaches to reduce treatment burden.^9^

Exercise is emerging as a potential alternative to ACT, acting to enhance mucociliary clearance and facilitate secretion removal.^15^ Structured exercise is already a recommended component of ‘usual care’ for pwCF,^5^ with aerobic exercise capacity being an important survival predictor.^16 17^ While current usual care recommendations advocate exercise *in addition to* chest physiotherapy,^5^ pwCF are already changing their approach based on preference to either incorporate exercise into their airway clearance regimens^18^ or to omit chest physiotherapy if they exercise.^19^ This preference underscores the need to investigate exercise as a primary ACT, engaging with patient priorities to address the question of whether exercise could replace chest physiotherapy, a priority highlighted by the James Lind Alliance.^20^

Whether exercise alone can serve as an effective ACT remains unknown.^15^ A recent UK Delphi consensus^21^ defined a bespoke exercise as an ACT (ExACT) intervention, proposing it as a potential substitute for chest physiotherapy during stable periods, that warranted further study. ExACT involves >20 minutes of aerobic exercise inducing deep breathing^21 22^ and is performed by predominantly weight-bearing exercise types that incorporate vibration.^22^ Huffing and coughing (forced expiratory techniques) are integral to ExACT,^23^ with pre- and post-session breaths to check for, and remove mucus. ExACT is self-administered, promoting independence and flexibility.^24^ Combining daily exercise and ACT recommendations as ‘ExACT’ could yield significant time savings and health benefits. With the widespread availability of HEMT, such as Elexacaftor/Tezacaftor/Ivacaftor (ETI), realigning treatment burden is now a top priority in CF care.^6^ High-quality evidence is needed to guide clinical practice.

### Aims

The ‘Exercise as an Airway Clearance Technique in CF (ExACT-CF)’ randomised pilot trial aimed to test the primary hypothesis of the feasibility and acceptability of the ExACT intervention when compared with usual care, together with safety and early signals of efficacy.

## Methods

The study was designed to establish the feasibility and acceptability of recruitment (target n=50), randomisation and retention for a 28 day trial comparing usual care with ExACT. The acceptability and feasibility of the ExACT intervention, for participants and trial delivery staff, were also established. Process evaluation, including intervention fidelity assessment, was undertaken, along with testing of the feasibility of collecting healthcare service resource use. Criteria for progression to a definitive trial were preset for recruitment, retention and adherence using traffic light criteria^22^ (table 1); green denoting ‘proceed’ - no concerning issues threatening success of definitive trial, amber denoting modification required for a larger trial, and red denoting likely non-progression.

**Table 1:** Performance against pre-defined trial progression criteria

	Green	Amber	Red
Recruitment/randomisation Number randomised/number approached	≥ 60%	30–59%	< 30%
Stop-go study outcome	48/117, 41% (amber)		
Retention Number providing primary clinical outcome data/number randomised	> 85%	70–84%	< 70%
Stop-go study outcome	45/48, 92% (green)		
Adherence Number undertaking satisfactory* airway clearance/number randomised	≥ 80%	60–79%	< 60%
Stop-go study outcome	29/48, 60% (amber)		

* Adherence was defined as undertaking airway clearance on ≥ 5 days out of 7 for both the usual care chest physiotherapy arm and the exercise as an airway clearance technique (ExACT) intervention arm. Details regarding the predefined trial progression criteria are available in the published protocol.^22^

A brief methods overview is provided in accordance with guidance for reporting pilot trials,^25^ with further details in the published protocol.^22^ The safety of stopping chest physiotherapy and replacing it with ExACT was also evaluated through changes in lung function, rates of pulmonary exacerbations and adverse events over the 28 day trial period. Additionally, a variety of candidate primary and secondary outcomes (physical activity (PA), treatment burden, quality of life (QoL) and sleep outcomes) were assessed for signals of efficacy, as well as for their associated burden, levels of completeness and estimates of outcome variance (to inform the sample size of a definitive trial).

In brief, lung clearance index (LCI_2.5_) was measured at baseline and day 28 by nitrogen-multiple breath washout (N_2_-MBW, Exhalyzer-D device, EcoMedics) and most recent software (Spiroware V.3.3.1). Spirometry (Spirobank Smart, Intermedical UK, Kent, UK) was performed either in hospital or at home, at baseline and days 7, 14, 21 and 28. Data for forced expiratory volume in 1 s (FEV_1_) and forced vital capacity (FVC) were converted to % predicted values in accordance with Global Lung Initiative reference values.^26^

### Study design

A two-arm, parallel-group, dual-site randomised pilot trial, with mixed-methods process and economic evaluations, was conducted at two UK sites. Eligible participants were randomised to ExACT or usual care arms. Statistical analysis and health economics analysis plans were developed *a priori*. An embedded qualitative substudy (ExACT-Q) explored participant and trial staff experience, though these data will be predominantly presented elsewhere. Study duration (28 days) and the requirement for all participants to be on ETI were informed by our community involvement and in order to undertake the study safely.

### Participants

PwCF (≥10 years, FEV_1_ ≥40% predicted; table 2), stable on ETI, were eligible, with target recruitment *n*=50.^22^ Two participants withdrew prior to randomisation. Comprehensive inclusion/exclusion criteria are published.^22^ Potential participants were screened by clinicians and research nurses at consecutive clinics. Clinical records were reviewed to confirm eligibility, and potential participants were approached in person during routine clinic visits or via telephone/email. Clinical stability was assessed by the participant’s treating clinician. Participants were not excluded due to any pathogen, if clinically stable. All participants provided fully informed written consent, with parental consent and participant assent for childhood participants.

**Table 2:** Baseline participant demographics at randomisation

	Usual care	ExACT	Overall
Randomised participants, n	24	24	48
Age, years	19.1 (10.4)	21.8 (12.8)	20.5 (11.6)
Adults ≥ 16 years, n	11	14	25
Children <16 years, n	13	10	23
Sex (M/F), n	14/10	14/10	28/20
Height, m	161.7 (15.3)	162.8 (13.6)	162.3 (14.3)
Weight, kg	53.5 (16.6)	63.0 (18.9)	58.2 (18.2)
BMI, kg/m^2^	20.1 (3.9)	23.2 (4.5)	21.7 (4.4)
Ethnicity, n (%)			
White – UK	22 (92)	22 (92)	44 (92)
White – European	2 (8)	2 (8)	4 (8)
CFTR genotype, n			
F508del/F508del	15	13	28
F508del/other	9	11	20
Dornase alfa, n (%)	20 (83)	13 (54)	33 (69)
Hypertonic saline, n (%)	1 (4)	2 (8)	3 (6)
Nebulised antibiotics, n (%)	8 (33)	5 (21)	13 (27)
Colistin	3 (13)	2 (8)	5 (10)
Tobramycin	4 (17)	2 (8)	6 (13)
Aztreonam	1 (4)	0 (0)	1 (2)
Meropenem	0 (0)	1 (4)	1 (2)
Prophylactic antibiotics, n (%)	7 (29)	6 (25)	13 (27)
Azithromycin	5 (21)	3 (13)	8 (17)
Flucloxacillin	2 (8)	1 (4)	3 (6)
Cotrimoxazole	0 (0)	1 (4)	1 (2)
Coamoxiclav	0 (0)	1 (4)	1 (2)
CFRD, n (%)	4 (17)	5 (21)	9 (19)
Pancreatic insufficient, n (%)	22 (92)	20 (83)	42 (88)
LCI_2.5_	8.3 (3.2)	7.9 (2.0)	8.3 (3.2)
FEV_1_, % predicted	84.1 (19.9)	90.2 (16.0)	87.1 (18.1)
FVC, % predicted	92.3 (14.2)	95.9 (11.2)	94.1 (12.7)

Values are means (SDs) unless otherwise stated.

Two participants withdrew prior to randomisation.

BMI, body mass index; CFRD, cystic fibrosis-related diabetes; CFTR, cystic fibrosis transmembrane conductance regulator; ExACT, exercise as an airway clearance technique; FEV_1_, forced expiratory volume in 1 s; FVC, forced vital capacity; LCI, Lung Clearance Index.

### Intervention

In accordance with the Medical Research Council’s framework for the development and evaluation of complex interventions,^27^ and considerable Patient and Public Involvement and Engagement (PPIE),^21^ we designed the ExACT intervention.^22^ Briefly, ExACT is aerobic exercise lasting > 20 minutes, at an intensity sufficient to be out of breath, and into which are incorporated forced expiratory techniques, specifically huffs and coughs. Participants were asked to stop all chest physiotherapy ACTs and instead undertake ExACT at least once a day for 28 days. All other CF treatments, including mucolytic and mucoactive agents, were continued. This was unsupervised following initial instruction.

### Usual care

Participants continued chest physiotherapy ACTs at least once a day. Exercise and PA were encouraged and advised to be maintained, but no specific instructions were provided. A chest physiotherapy refresher was offered, given knowledge that adherence and quality of chest physiotherapy are frequently suboptimal.^28^

### Adherence

As outlined in our published protocol,^22^ adherence was monitored in both study arms using a combination of self-report and objective measures. Participants recorded daily sessions via a purpose-built, participant-facing study database, including details of both ExACT and traditional chest physiotherapy ACTs, as well as any additional PA. They also rated their perceived exercise intensity. In addition, participants were instructed to start and stop each session – whether ExACT or chest physiotherapy ACTs – using their Garmin device, enabling capture of session duration and physiological response (eg, heart rate). Informed by our PPIE, adherence was defined as undertaking airway clearance on ≥ 5 days in each week for both arms of the trial. These procedures were designed to assess the feasibility and acceptability of capturing adherence through a mixed-methods approach across both arms of the trial.

### Randomisation and blinding

The randomisation sequence was computer generated within a bespoke study database, hosted by Edinburgh Clinical Trials Unit. Randomisation was at the individual level, with a 1:1 allocation ratio, and minimised (with a random element) by age (< 20 and > 20 years), lung function (FEV_1_ < 70% predicted and >70% predicted^26^) and baseline device-measured (Garmin Vivosmart 4) PA (< 3 and > 3 hours of moderate to vigorous intensity physical activity (MVPA) / week). The randomisation algorithm for each participant (other than the first) allocated them with a probability of 0.8 to the group that minimised the difference between the two arms of the trial with respect to the minimisation variables. The study was, in effect, ‘open label’, with neither participant nor treating clinicians blinded to the study allocation.

### Outcome measures

#### Feasibility

Feasibility was assessed through recruitment, retention and adherence over 28 days, against pre-defined criteria for progression to a definitive trial (table 1), as laid out in the study protocol.^22^ More specifically, green (good to proceed); amber (proceed with modifications); and red (not recommended to proceed) progression criteria were devised for the proportion of pwCF randomised, completing the study and submitting necessary data at 28 days, and adhering to the intervention. In addition, we quantified (by exit questionnaire) the proportion that would choose the ExACT intervention over usual care (chest physiotherapy) in the future.

#### Safety and efficacy

The safety and potential efficacy of replacing chest physiotherapy with ExACT were assessed via adverse events and change from baseline to day 28 in spirometry (FEV_1_), LCI_2.5_ by N_2_-MBW and rates of pulmonary exacerbations (defined as increased respiratory symptoms that required antibiotic treatment).

#### Health economic evaluation

A health economic evaluation was conducted in accordance with best practice guidance for clinical trials,^29^ in order to pilot data collection methods. Descriptive statistics and an assessment of the completeness of self-report surveys/questionnaires, along with preliminary cost-utility estimates, were provided. Data are presented in accordance with guidance for Consolidated Health Economic Evaluation Reporting Standards.^30^ Where suitable/deemed viable, exploratory, within-trial analysis was undertaken to provide provisional cost-utility estimates and quality-adjusted life years (QALYs) and understand the main cost-effectiveness drivers.^31^

#### Additional outcomes

Other secondary clinical outcomes included changes in PA, sleep, mood, QoL and treatment burden. Fidelity was assessed through independently scored role-play videos of selected trial staff explaining the study and intervention (online supplemental table S1), as well as intensity of sessions being recorded via activity monitors.

### Data analysis

**Figure 1:**
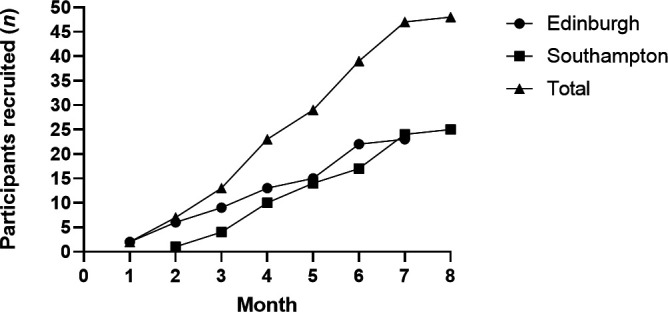
Recruitment trajectory for the ExACT-CF trial for the overall cohort and by site. ExACT-CF, Exercise as an Airway Clearance Technique in CF; FEV_1_, forced expiratory volume in 1 s.

As this was a feasibility pilot trial, analysis was primarily descriptive, with participant flow, screening, recruitment and attrition rates summarised with exact 95% CI (figure 1) displayed alongside. Means and SDs were reported for exploratory outcomes at baseline and 7, 14, 21 and 28 days for each group. Intention-to-treat analyses were performed. To facilitate power calculations of a future trial, linear regression models were fitted on outcome variables regressed on treatment adjusting for baseline. Where appropriate, log transformations were performed to adhere to the assumption of normality. Models adjusting for baseline and minimisation variables gave similar results (data not shown).

## Results

**Figure 2:**
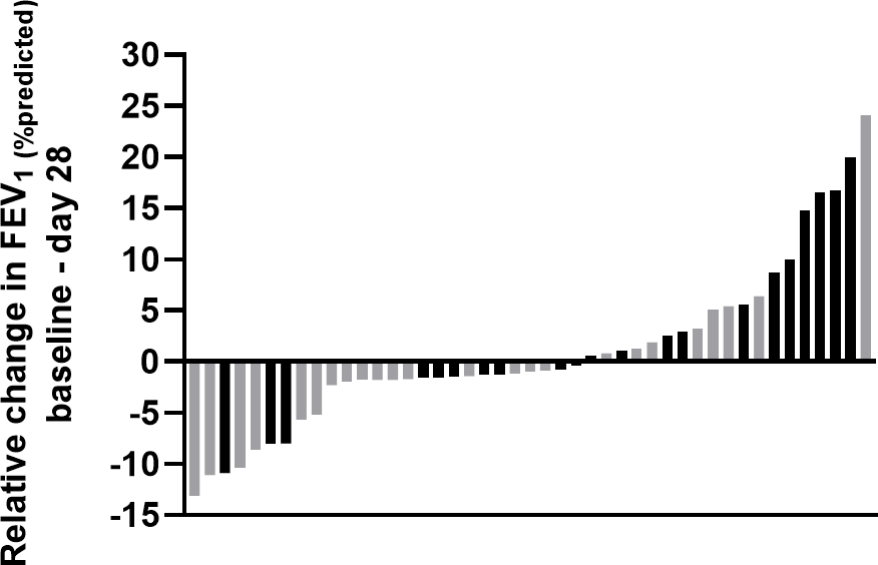
Waterfall plot delineating change in FEV_1_ for participants in the ExACT (● black) and chest physiotherapy (● grey) trial arms over 28 days. ExACT, exercise as an airway clearance technique; FEV_1_, forced expiratory volume in 1 s.

Recruitment took place from February to September 2023. The baseline demographics of study participants in each arm are displayed in table 2. Baseline airway clearance is summarised in table 3. Recruitment was on target and on time (figure 2).

**Table 3:** Reported methods of airway clearance at randomisation (n = 48)

	Usual care (n=24)	ExACT (n=24)	Overall (n=48)
Chest physiotherapy (n, %)	21/24, 88%	16/24, 67%	37/48, 77%
Chest physiotherapy, type (n, %)			
PEP mask	10/21, 48%	4/16, 25%	14/37, 38%
Aerobika	4/21, 19%	4/16, 25%	8/37, 22%
Acapella	3/21, 14%	2/16, 13%	5/37, 14%
Active cycle breathing techniques	1/21, 5%	1/16, 6%	2/37, 5%
Autogenic drainage	4/21, 19%	6/16, 38%	10/37, 27%
Exercise (n, %)	10/24, 42%	10/24, 42%	20/48, 42%
Mixture of exercise and chest physiotherapy (n, %)	8/24, 33%	6/24, 25%	14/48, 29%
Only undertaking exercise as airway clearance (n, %)	3/24, 13%	6/24, 25%	9/48, 19%
No regular airway clearance being undertaken (n, %)	0/24, 0%	2/24, 8%	2/48, 4%

No participant reported using high-frequency chest wall oscillation devices, cough assist or non-invasive ventilation devices for airway clearance.

ExACT, exercise as an airway clearance technique.

### Feasibility and acceptability of recruitment, randomisation, retention, and adherence to the ExACT intervention, processes and outcome measures

We screened/approached 117 pwCF, of whom 48 (41% of those approached) were randomised (figure 1); amber for trial progression (table 1). One individual became ineligible (FEV_1_< 40% predicted) between consent and randomisation, and another withdrew consent. In total, 45/48 (92%) randomised participants provided data at day 28, supporting progression (green) to a definitive trial. Adherence through 28 days for the ExACT intervention was 60% (amber for trial progression; table 1). Adherence data for both the ExACT intervention and usual care chest physiotherapy ACTs are presented in online supplemental table S5. It is noted that in those completing the study, adherence to ExACT exceeded that of usual care at all time points.

### Safety and clinical impact of replacing routine chest physiotherapy with ExACT

The study identified no safety concerns with regard to the impact of stopping chest physiotherapy on lung health over a 28-day period. LCI_2.5_ was similar in both groups at each time point (table 4). Mean (SD) changes in FEV_1_ (% predicted) from baseline to day 28 were +2.1 (6.6) in the ExACT arm and −0.8 (5.5) in the usual care arm. Exploratory linear regression models suggested trends towards estimated mean (95% CI) between group (ExACT vs usual care) differences for change in both LCI_2.5_ and FEV_1_ from baseline to day 28, in favour of ExACT. Data for LCI_2.5_ showed a relative difference (ExACT/usual care) of 0.97 (95% CI 0.92 to 1.02—back-transformed results from analyses on log normal data), while FEV_1_ absolute difference (ExACT−usual care) was 3.2% predicted (95% CI −0.6% to 6.9%).

**Table 4:** Lung function and anthropometric changes from baseline to day 28 by treatment allocation

Parameter	Usual care (n=24)			ExACT (n=21)		
	Baseline	Day 28	Difference	Baseline	Day 28	Difference
LCI_2.5_	8.3 (3.2)	8.5 (3.3)	0.2 (0.8)	7.9 (2.0)	7.8 (1.9)	−0.1 (0.6)
FEV_1_, % predicted	84.1 (19.9)	83.3 (20.6)	−0.8 (5.5)	89.9 (17.2)	92.1 (16.6)	2.1 (6.6)
FVC, % predicted	92.3 (14.2)	90.0 (13.5)	−2.3 (5.8)	95.7 (11.8)	96.4 (12.4)	0.8 (7.5)
Height, m	1.62 (1.53)	1.62 (1.51)	0.0 (0.69)	1.62 (1.37)	1.63 (1.34)	0.0 (1.09)
Weight, kg	53.5 (16.6)	53.7 (16.3)	−0.2 (0.9)	62.3 (18.7)	61.9 (18.5)	0.3 (1.2)
BMI, kg/m^2^	20.1 (3.9)	20.1 (3.7)	−0.1 (0.4)	23.1 (4.5)	23.0 (4.6)	0.1 (0.4)

Values are means (SDs) for complete cases, unless otherwise stated.

BMI, body mass index; ExACT, exercise as an airway clearance technique; FEV_1_, forced expiratory volume in 1 s; FVC, forced vital capacity; LCI, Lung Clearance Index.

**Figure 3:**
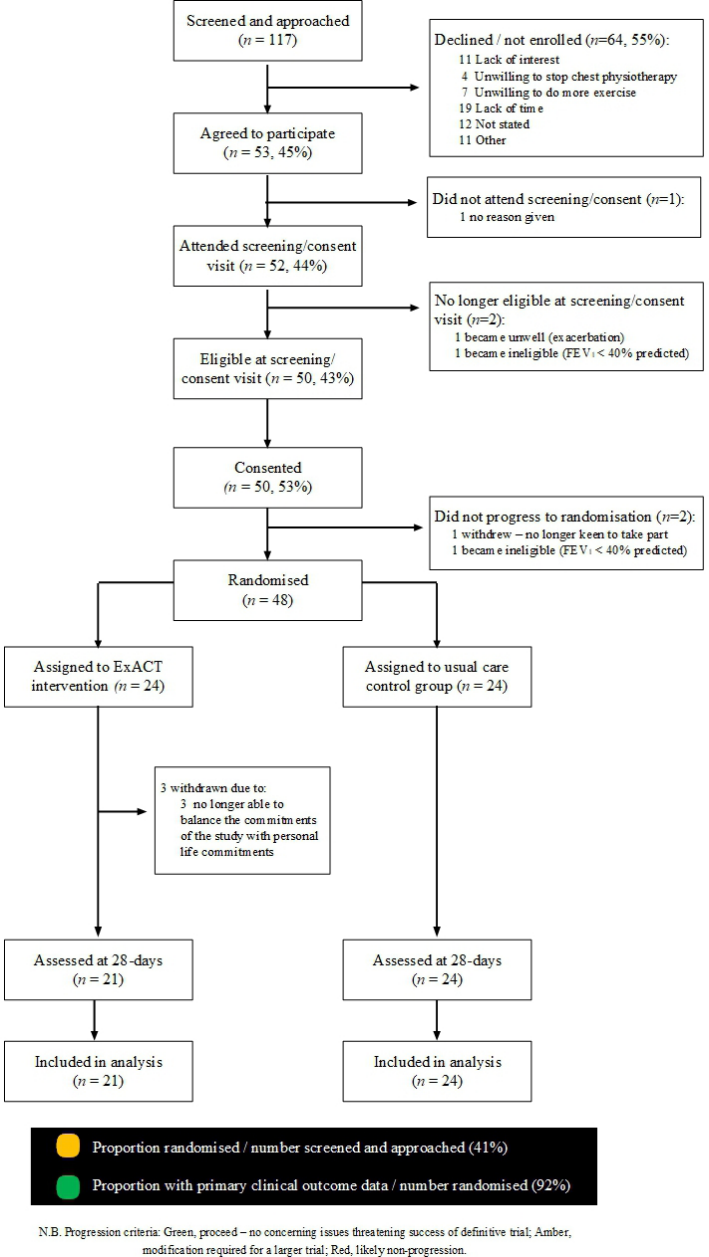
CONSORT flow diagram for the ExACT-CF study. CONSORT, Consolidated Standards of Reporting Trials; ExACT-CF, Exercise as an Airway Clearance Technique in CF.

Inter-individual variation was, however, noted (figure 2), with the majority of the observed changes being lower than the accepted within-subject variability (<8%) for FEV_1_.^32^ 11 of the 14 greatest observed falls in FEV_1_ (% predicted) were in the usual care group, while six of the seven biggest increases were in the ExACT group (figure 3). Post hoc analyses revealed similar height, weight and BMI over the 28-day period in both treatment allocation groups (table 4).

A total of 51 adverse events were recorded for 25 different trial participants, none of which were ‘Serious’ (table 5). There was a higher proportion of exercise-related injuries in the ExACT group, none being severe. Injuries included a bicycle fall, groin strain, painful coccyx related to a bicycle seat and pain in the knee, shin, hip and foot. All were managed with rest and/or simple analgesia, while five were coded as ‘possibly related’ and two ‘unrelated’. No participant had to stop ExACT and resume their previous ACT regimen. Self-reported weekly changes in sputum volume or colour are reported in online supplemental table S4.

**Table 5:** Summary of adverse events in the ExACT-CF pilot trial (n*=*25 experienced)

Adverse event	ExACT (n, number coded as possibly related to ExACT)	Usual care (n)
Respiratory		
Pulmonary exacerbation	2, 1	2
Antibiotic treatment for chest	4, 2	4
Cough	4, 0	5
Nasal congestion	1, 0	0
Sore throat	2, 0	0
Tight chest/breathlessness	2, 2	1
Coryza	1, 0	1
Hay fever	0, 0	1
Ear infection	1, 0	1
Sports injuries		
Knee pain	3, 3	0
Foot pain	1, 1	0
Shin pain	1, 0	0
Painful coccyx	1, 1	0
Groin pain	1, 1	0
Hip pain	1, 0	1
Other		
Hypoglycaemic episode	1, 1	0
Abdominal pain	1, 0	0
Fatigue	4, 1	2
Headache	1, 0	0
Fever	1, 0	0
Vomiting	1, 0	0
Nasal injury	1, 0	0

ExACT, exercise as an airway clearance technique; ExACT-CF, Exercise as an Airway Clearance Technique in CF.

### Additional outcomes

#### Physical activity

Exploratory analyses of device-measured PA revealed higher step counts at day 28 in the ExACT arm, although a potential between-group difference at baseline is noted. Although MVPA (table 6) at day 28 was similar between the groups, the ExACT arm increased by 60 minutes/week (mean) from baseline versus an increase of 5 minutes (mean) in MVPA for those receiving usual care. Habitual Activity Estimation Scale questionnaire data suggested that ExACT participants spent more time active across weekdays and weekends from baseline to day 28 with a reduction in activity over the 28 days in the usual care arm (table 6).

**Table 6:** Physical activity and sleep in the ExACT-CF study

Outcome measure	Data collection time point										
	Baseline		Day 7*			Day 14*		Day 21*		Day 28*	
	ExACT (n=24)	UC (n=24)	ExACT (n=22)	UC (n=24)	ExACT (n=21)		UC (n=24)	ExACT (n=21)	UC (n=24)	ExACT (n=21)	UC (n=24)
Device-based PA											
Total steps, steps/day	10 331 (5246)	8859 (3640)	10 646 (5249)	9196 (3773)		10 714 (4564)	9503 (5955)	11 198 (4936)	7945 (4058)	10 784 (6187)	8181 (3105)
MVPA, min/week	420 (350)	469 (310)	550 (398)	450 (363)		456 (232)	340 (308)	524 (316)	383 (346)	478 (347)	475 (551)
Sleep time, hours/day	8.2 (1.8)	7.9 (1.8)	8.0 (1.7)	8.2 (1.4)		8.0 (1.8)	8.3 (1.5)	7.9 (1.5)	8.2 (1.5)	8.1 (1.9)	7.9 (1.6)
PA data from patient-facing exercise diary											
Exercise sessions, sessions/week	–	–	9.1 (3.7)	4.5 (2.8)		8.1 (3.2)	3.9 (8.1)	7.8 (4.3)	4.1 (3.8)	7.5 (2.9)	3.3 (3.2)
Weekly PA, minutes/week	–	–	356 (213)	325 (220)		430 (651)	348 (200)	328 (268)	296 (280)	286 (168)	320 (235)
Time spent active or somewhat active (HAES questionnaire)											
Weekday, minutes/day	441.6 (168.0)	470.6 (184.6)	–	–		–	–	–	–	496.2 (160.8)	408.6 (218.0)
Weekend, minutes/day	439.4 (186.9)	483.9 (177.9)	–	–		–	–	–	–	477.7 (188.5)	397.4 (180.2)

* Postrandomisation. Values are expressed as numbers, means and (SD) as a function of trial arm and measurement time point for PA and sleep.

ExACT, exercise as an airway clearance technique; EXACT-CF, Exercise as an Airway Clearance Technique in CF; HAES, Habitual Activity Estimation Scale; MVPA, moderate to vigorous intensity physical activity; PA, physical activity; UC, usual care.

#### Treatment burden

No additional treatment burden at 28 days is suggested (online supplemental table S2), although Cystic Fibrosis Questionnaire-Revised (CFQ-R) treatment burden subscale scores increased in both study arms. More weekly sessions of exercise were undertaken when ExACT was used for airway clearance (table 6).

#### Sleep, mood and QoL

Sleep duration was similar between the groups over 28 days (table 5). Slight reductions in anxiety and depression scores were noted in both study arms (online supplemental table S2). Scores for the CFQ-R domains at baseline and day 28 are presented in online supplemental table S2, with QoL domains that were similar between groups over the 28-day trial period.

#### Health economics

Health-related QoL data (using the EuroQol 5-dimension 5-level questionnaire (EQ-5D-5L) questionnaire) were collected with high levels of completeness. No large differences between trial arms were seen in respect of healthcare utilisation, EQ-5D-5L scores or QALYs.

All clinical trial outcome data were collected with high levels of completeness through 28 days (online supplemental table S3). For each outcome measure, we exceeded the number of cases recommended for pilot studies to estimate outcome variance.^33^

### Acceptability of the ExACT intervention, study outcomes and aspects of trial delivery

#### Fidelity

The approach and explanation of the trial scored highly across both sites (online supplemental table S3). Explaining the ExACT intervention was less consistent, suggesting that additional resource (videos, a web-based resource for participants and further role-play) may be required in a larger trial. Central over-reading of LCI_2.5_ data scored 44/45 (98%) as successful in Edinburgh and 46/49 (94%) in Southampton, with an overall acceptability rating of 96%.

#### Preference

In total, 44 participants completed the end of study questionnaire establishing their preferred ACT going forward. Only three people (7%, 1 ExACT) chose chest physiotherapy as their preferred ACT, with 12 (27%, 6 ExACT) selecting ExACT only, and 29 (66%, 13 ExACT) selecting interchangeable use of ExACT and chest physiotherapy.

#### Qualitative interviews

In this embedded study, 32 online/phone interviews were conducted with participants receiving ExACT (*n*=5) and usual care (*n*=5), parents/caregivers (*n*=5), decliners/dropouts (*n*=5) and health professionals (*n*=12). Two main themes emerged: facilitators and barriers to participation and trial delivery, and appetite for airway clearance regimes other than daily chest physiotherapy. Not a single interviewee wished to perform chest physiotherapy as their only ACT. The data confirmed the acceptability of most trial processes and the intervention, while identifying areas for improvement to enhance a definitive trial. Qualitative data will be published separately (*under review*).

## Discussion

The ExACT-CF pilot feasibility trial represents the first dual-site randomised controlled pilot trial to test the feasibility and acceptability of recruiting pwCF, stable on ETI, into a trial examining exercise as a form of airway clearance to replace traditional ACT, to inform whether a definitive randomised controlled trial focused on establishing efficacy and safety is possible. Meeting all pre-stated safety and feasibility criteria,^22^ this trial lays a strong foundation for a larger, definitive study. No clinical respiratory safety signals were identified in those who stopped traditional ACTs over 28 days in this feasibility trial. The findings suggest, from LCI_2.5_ and FEV_1_ changes over 28 days, that ExACT is at least as good as usual care, although signals towards efficacy of ExACT now warrant further investigation. Feasibility data demonstrate that pwCF are receptive to trials exploring ExACT, showing acceptable recruitment and retention, high levels of adherence and promising preliminary safety data.

The ExACT intervention, trial design and processes were well received by both participants and staff. Minimal participant burden and high outcome measure completion further support the acceptability of ExACT as an alternative to chest physiotherapy. The trial also effectively rehearsed health economic evaluation and trial governance procedures. Diary entries noted different levels of PA from that measured directly (using Garmin device) and suggest that cross-validation with a widely used measure (accelerometry) may be important for a larger trial. Qualitative feedback from participants and healthcare professionals indicated that the ExACT intervention was safe, enjoyable and less time-consuming than chest physiotherapy. While full findings will be reported separately, early insights highlight that many found integrating daily exercise feasible and flexible, though some faced challenges with routine or fatigue—suggesting the need for realistic adherence targets in future trials.

Exercise-related injuries were more commonly reported in pwCF undertaking ExACT (table 4). These were generally mild and self-resolving; however, they would require a modified training approach within a definitive trial to reduce this safety signal. With some modifications, progressing to a larger, more pragmatic trial, assessing the efficacy and cost-effectiveness of the ExACT intervention is feasible and justified.

In line with our published Delphi consensus,^21^ we demonstrated that a sufficient number of pwCF were willing to be randomised into an exercise-based airway clearance trial. However, competing CF trials impacted recruitment. Our study highlights the importance of effective collaboration with stakeholders, including robust PPIE mechanisms. Both trial sites were members of the UK CF Trust Clinical Trials Accelerator Platform, with ExACT-CF being one of the top-recruiting trials in their portfolio despite involving only two sites. A definitive trial will seek to ensure equitable access for pwCF across a wider range of sites.

This trial enabled us to evaluate the ExACT intervention and optimise the design for a definitive study. Patient preference from exit questionnaires and qualitative interviews demonstrated strong support for ExACT as a treatment modality—either stand-alone or interchangeable with usual care. The need for a pragmatic trial to confirm that ExACT is safe and non-inferior to usual care is therefore suggested, facilitating the integration of exercise into the airway clearance toolkit for pwCF. Further work to evaluate the safety, acceptability and efficacy of ExACT in other causes of bronchiectasis^34^ should also be considered.

The ExACT-CF feasibility study has several limitations. First, it was conducted across two UK centres (Edinburgh and Southampton) only, which may limit the generalisability of the findings. Second, the study exclusively included patients established on ETI (guided by our intervention development work with physiotherapists, pwCF and their families, and clinicians^21^), potentially affecting the applicability of results to all pwCF. However, this criterion was implemented to ensure participant stability and homogeneity within this small-scale feasibility trial. Third, the study was not powered to assess clinical efficacy; thus, any observed differences in clinical outcomes should be interpreted with caution. This aligns with the primary aim of feasibility studies, which is to assess the practicality of study procedures rather than to evaluate treatment effects. Additionally, the open-label design, while common in feasibility studies, may introduce bias, although blinding was not feasible given the nature of the intervention. Lastly, while adherence to the intervention was monitored, adherence to concurrent therapies, such as mucolytic and mucoactive agents and antibiotics, was not assessed, though broadly similar baseline usage was reported (table 2). Future studies should consider monitoring these factors, as changes in airway clearance regimens could influence adherence to other treatments, potentially impacting clinical outcomes.

This pilot trial identified areas needing refinement in design and management, essential for optimising the effectiveness of the ExACT intervention in future trials. Given the adherence being amber (60%), a more rigorous application of the behaviour change wheel^35^ and techniques such as motivational interviewing, which have shown promise in enhancing engagement and adherence to PA in pulmonary rehabilitation,^36^ are key considerations. Furthermore, incorporating injury prevention strategies and providing enhanced training resources for participants and trial staff are important areas for improvement.

The flexible, home-based delivery of trial interventions and some study visits was well received and aligns with prior qualitative insights from adults with CF, who expressed a preference for PA interventions that are enjoyable, foster autonomous motivation, have personal outcomes and can be performed at home.^37^ This highlights opportunities to leverage digital health technologies to support pwCF.^38^ Participant and staff experiences, along with lessons learnt from this trial, will be detailed in a forthcoming qualitative article.

Recent withdrawal studies, such as CF-STORM and SIMPLIFY,^39^ have focused on reducing treatment burden. However, whether chest physiotherapy can be safely modified or discontinued in the era of HEMT remained unclear.^40^ Our adaptive approach to realign ACTs to periods of clinical stability and instability may provide patients with an efficient method of self-care. Preliminary findings from the embedded health economic evaluation within the ExACT-CF pilot trial suggest that ExACT may be deliverable without significant additional cost, while the broader health benefits of structured aerobic exercise,^41^ such as maintaining health in an ageing CF population,^5^ may be being accrued.

ExACT aligns with the European CF Society Standards of Care for Maintaining Health in PwCF,^5^ which recently recognised exercise as a viable option in personalised, preference-based ACT regimens. While this validation of ExACT is cautiously welcomed, it is recognised that there is not yet a definitive trial to support such a statement.

Given the burden for pwCF who are currently being asked to undertake both ACT and exercise, ExACT may simplify treatment burden by combining both exercise and ACT together. Notably, although weekly exercise time was similar between groups, ExACT participants engaged in more moderate to vigorous-intensity exercise, likely conferring greater long-term fitness and health benefits.

A larger, definitive trial is needed to rigorously test the efficacy, cost-effectiveness and safety of the ExACT intervention. Insights from this could influence clinical practice and potentially extend to other respiratory diseases, for example, primary ciliary dyskinesia and other causes of bronchiectasis. Bronchiectasis, affecting > 2 00 000 people in the UK,^42^ poses a significant burden,^43^ and similar to CF also poses challenges with long-term adherence to ACT and exercise.^34^ This could lead to a programme of research that may offer a more manageable and effective approach to airway clearance applicable across chronic suppurative lung diseases of differing aetiology.

In conclusion, ExACT is feasible, acceptable and appears safe for pwCF over 28 days. Its adoption as a recommended ACT, alone or in combination with chest physiotherapy, could enhance CF management by increasing flexibility and reducing treatment burden, and aligning with the needs and preferences of pwCF. A definitive trial is needed to confirm its clinical effectiveness and cost-effectiveness, supporting broader integration into CF care and potentially other chronic respiratory conditions. This work addresses key priorities for pwCF and has the potential to significantly influence clinical practice.

## Supplementary material

10.1136/thorax-2025-223080 online supplemental file 1

## Data Availability

Data are available upon reasonable request.
