# Correction: *Safety, feasibility and efficacy of exercise as an airway clearance technique in cystic fibrosis: a randomised pilot feasibility trial*

**DOI:** 10.1136/thorax-2025-223080corr1

**Published:** 2026-02-16

Urquhart DS, Taylor E, Cunningham S ExACT-CF Study Group*, et al* Safety, feasibility and efficacy of exercise as an airway clearance technique in cystic fibrosis: a randomised pilot feasibility trial. *Thorax* 2026;81:140–149.

Figure legends were placed with the incorrect figures. These have now been added correctly in the online PDF and HTML versions.

