# Supplementary material for: Safety, feasibility and efficacy of exercise as an airway clearance technique in cystic fibrosis: a randomised pilot feasibility trial
Source: Thorax. 2025 Oct 1;81(2):e223080. doi: 10.1136/thorax-2025-223080 (PMC12911658; doi:10.1136/thorax-2025-223080)
Supplement: online supplemental file 1 [file thorax-81-2-s001.docx]

**ONLINE SUPPLEMENTARY MATERIAL**

**Table S1.** Fidelity assessment across roles and trial sites

|  | **Edinburgh** | | | **Southampton** | |
| --- | --- | --- | --- | --- | --- |
|  | **PI** | **Research Fellow** | **Research Nurse** | **PI** | **Research Nurse** |
| **Approach/explanation of trial,** *n, %* | 8/8 (100%) | 4/8  (50%) | 8/8 (100%) | 6.5/8 (81%) | 7/8  (88%) |
| **ExACT Intervention,** *n, %* | 2/9 (22%) | 9/9  (100%) | 9/9  (100%) | 5/9  (56%) | 5.5/9 (61%) |

N.B. ExACT, exercise as an airway clearance technique; PI, principal investigator.

**Table S2.** Quality of life, mood and treatment burden at baseline and day 28 by treatment allocation.

|  | **Usual Care** | | | **ExACT** | | |
| --- | --- | --- | --- | --- | --- | --- |
| **Parameter** | **Baseline** | **Day 28** | **Difference** | **Baseline** | **Day 28** | **Difference** |
| ***Quality of life (CFQ-R Domain)*** | | | | | | |
| **Respiratory** | 94 (9) | 91 (18) | -3 (16) | 91 (10) | 88 (12) | -2 (14) |
| **Health** | 88 (14) | 84 (18) | -4 (15) | 85 (19) | 85 (16) | 1 (19) |
| **Digestive** | 88 (14) | 89 (13)) | 2 (8) | 86 (19) | 87 (16) | 1 (14) |
| **Weight** | 74 (38) | 75 (37) | 1 (18) | 93 (22) | 94 (23) | 0 (11) |
| **Eating** | 89 (21) | 90 (19) | 1 (11) | 90 (27) | 91 (21) | -1 (15) |
| **Vitality** | 73 (14) | 75 (15) | 3 (12) | 67 (17) | 71 (16) | 4 (16) |
| **Emotion** | 89 (13) | 91 (11) | 2 (9) | 90 (12) | 90 (11) | -2 (8) |
| **Body** | 85 (23) | 83 (24) | -1 (11) | 84 (18) | 78 (24) | -6 (25) |
| **Physical** | 93 (9) | 90 (11) | -4 (7) | 93 (11) | 93 (14) | 0 (10) |
| **Treatment** | 77 (15) | 82 (14) | 5 (11) | 77 (20) | 79 (21) | 2 (17) |
| **Social*** | 82 (14) | 81 (14) | -1 (9) | 87 (10 | 87 (10) | -2 (11) |
| **Role*** | 90 (20) | 91 (14) | 1 (10) | 91 (15) | 94 (13) | 4 (22) |
| **School^#^** | 84 (13) | 93 (7) | 9 (13) | 73 (22) | 82 (12) | 4 (18) |
| ***Mood (HADS)*** | | | | | | |
| **Depression** | 1.9 (2.6) | 1.6 (2.0) | -0.3 (1.4) | 2.4 (3.1) | 1.8 (2.5) | -0.5 (1.8) |
| **Anxiety** | 5.0 (4.2) | 3.9 (3.4) | -0.9 (3.2) | 5.4 (5.7) | 3.9 (4.8) | -0.6 (2.6) |

* Participants who completed the adult/adolescent CFQ 14+ questionnaire only; ^#^Participants who completed the parent/caregiver CFQ (6-13) questionnaire only. N.B. Data are displayed as means (standard deviations) unless otherwise stated. N.B. CFQ-R, cystic fibrosis questionnaire revised

**Table S3.** Completeness of trial clinical outcome measures.

|  | **Data collection timepoint** | | | | | | | | | |
| --- | --- | --- | --- | --- | --- | --- | --- | --- | --- | --- |
|  | **Baseline** | | **Day 7*** | | **Day 14*** | | **Day 21*** | | **Day 28*** | |
| **Outcome measure** | **ExACT** | **Usual Care** | **ExACT** | **Usual Care** | **ExACT** | **Usual Care** | **ExACT** | **Usual Care** | **ExACT** | **Usual Care** |
| **Device-based physical activity, *n*, %** | 24/24 | 24/24 | 22/24 | 24/24 | 21/24 | 24/24 | 21/24 | 24/24 | 19 (79%) | 23 (96%) |
| **Spirometry, *n*, %** | 24/24 | 24/24 | 21/24 | 24/24 | 21/24 | 24/24 | 21/24 | 23/24 | 21/24 (88%) | 24/24 (100%) |
| **LCI, *n*, %)** | 23/24 | 24/24 | 21/24 | 24/24 | 21/24 | 24/24 | 21/24 | 23/24 | 20/24 (83%) | 24/24 (100%) |

*Post-randomisation. Values are expressed as means (SD) unless otherwise stated. N.B. ExACT, exercise as an airway clearance technique; LCI, lung clearance index; *n*, number.

**Table S4.** Self-reported changes in sputum volume or colour at baseline to day 28 by treatment allocation including *post hoc* per-protocol analyses.

| **Treatment** | | | |
| --- | --- | --- | --- |
|  | **Usual Care**  **(*n* = 24)** | **ExACT**  **(*n* = 24)** | **All**  **(*n* = 48)** |
| **Baseline** |  |  |  |
| **Yes** | 2 (8%) | 2 (8%) | 4 (8%) |
| **No** | 22 (92%) | 22 (92%) | 44 (92%) |
| **Day 7** |  |  |  |
| **Yes** | 1 (4%) | 2 (9%) | 3 (7%) |
| **No** | 23 (96%) | 20 (91%) | 43 (93%) |
| **Missing** |  | 2 | 2 |
| **Day 14** |  |  |  |
| **Yes** | 0 | 4 (19%) | 4 (9%) |
| **No** | 24 (100%) | 17 (81%) | 41 (91%) |
| **Missing** |  | 3 | 3 |
| **Day 21** |  |  |  |
| **Yes** | 2 (8%) | 4 (19%) | 6 (13%) |
| **No** | 22 (92%) | 17 (81%) | 39 (87%) |
| **Missing** |  | 3 | 3 |
| **Day 28** |  |  |  |
| **Yes** | 2 (8%) | 4 (19%) | 6 (13%) |
| **No** | 22 (92%) | 17 (81%) | 39 (87%) |
| **Missing** |  | 3 | 3 |

**Table S5.** Adherence to airway clearance during the ExACT-CF pilot trial.

| **Timepoint** | **ExACT** | **Usual Care** | **Overall** |
| --- | --- | --- | --- |
| **Adherence to airway clearance on ≥ 5 days of 7 days (all participants)** | | | |
| **Day 7** | 19/24 (79%) | 18/24 (75%) | 37/48 (77%) |
| **Day 14** | 16/24 (67%) | 16/24 (67%) | 32/48 (67%) |
| **Day 21** | 17/24 (71%) | 18/24 (75%) | 35/48 (75%) |
| **Day 28** | 15/24 (63%) | 14/24 (58%) | 29/48 (60%) |
| **Adherence to airway clearance on ≥ 5 days of 7 days (45 participants who completed the study only)** | | | |
| **Day 7** | 19/22 (86%) | 18/24 (75%) | 37/46(80%) |
| **Day 14** | 16/21 (76%) | 16/24 (67%) | 32/45 (71%) |
| **Day 21** | 17/21 (81%) | 18/24 (75%) | 36/45 (80%) |
| **Day 28** | 15/21 (71%) | 14/24 (58%) | 29/45 (64%) |
| **Weekly airway clearance sessions** | | | |
| **Day 7** | 7.4 (2.4) | 6.5 (2.5) | - |
| **Day 14** | 7.2 (2.9) | 6.2 (3.1) | - |
| **Day 21** | 6.9 (3.5) | 6.5 (3.3) | - |
| **Day 28** | 6.9 (2.9) | 5.8 (3.0) | - |
|  |  |  |  |

N.B. ExACT, exercise as an airway clearance technique.
